# Enhancement of Electrochemical Performance of LiMn_2_O_4_ Spinel Cathode Material by Synergetic Substitution with Ni and S

**DOI:** 10.3390/ma9050366

**Published:** 2016-05-13

**Authors:** Monika Bakierska, Michał Świętosławski, Marta Gajewska, Andrzej Kowalczyk, Zofia Piwowarska, Lucjan Chmielarz, Roman Dziembaj, Marcin Molenda

**Affiliations:** 1 Faculty of Chemistry, Jagiellonian University, Ingardena 3, Krakow 30-060, Poland; monika.bakierska@gmail.com (M.B.); m.swietoslawski@gmail.com (M.Ś.); kowalczy@chemia.uj.edu.pl (A.K.); zpiwowa@gmail.com (Z.P.); lucjanchmielarz@gmail.com (L.C.); dziembaj@chemia.uj.edu.pl (R.D.); 2 Academic Centre for Materials and Nanotechnology, AGH University of Science and Technology, Mickiewicza 30, Krakow 30-059, Poland; marta.gajewska@agh.edu.pl

**Keywords:** Li-ion battery, cathode material, LiMn_2_O_4_ spinel, co-doping, electrochemical performance

## Abstract

Nickel and sulfur doped lithium manganese spinels with a nominal composition of LiMn_2−*x*_Ni*_x_*O_4–*y*_S*_y_* (0.1 ≤ *x* ≤ 0.5 and *y* = 0.01) were synthesized by a xerogel-type sol-gel method followed by subsequent calcinations at 300 and 650 °C in air. The samples were investigated in terms of physicochemical properties using X-ray powder diffraction (XRD), transmission electron microscopy (EDS-TEM), N_2_ adsorption-desorption measurements (N_2_-BET), differential scanning calorimetry (DSC), and electrical conductivity studies (EC). Electrochemical characteristics of Li/Li^+^/LiMn_2−*x*_Ni*_x_*O_4–*y*_S*_y_* cells were examined by galvanostatic charge/discharge tests (CELL TEST), electrochemical impedance spectroscopy (EIS), and cyclic voltammetry (CV). The XRD showed that for samples calcined at 650 °C containing 0.1 and 0.2 mole of Ni single phase materials of Fd-3m group symmetry and nanoparticles size of around 50 nm were obtained. The energy dispersive X-ray spectroscopy (EDS) mapping confirmed homogenous distribution of nickel and sulfur in the obtained spinel materials. Moreover, it was revealed that the adverse phase transition at around room temperature typical for the stoichiometric spinel was successfully suppressed by Ni and S substitution. Electrochemical results indicated that slight substitution of nickel (*x* = 0.1) and sulfur (*y* = 0.01) in the LiMn_2_O_4_ enhances the electrochemical performance along with the rate capability and capacity retention.

## 1. Introduction

The search for low weight, high energy and power density lithium-ion batteries (LIBs) has increased in recent years due to a growing demand for energy storage in the field of large scale applications (e.g., hybrid electric vehicles, electric vehicles (xEV), and stationary energy storage systems (ESS)) [1,2,3].

One of the most attractive cathode materials for rechargeable LIBs is lithium manganese oxide spinel (LiMn_2_O_4_, LMO). In comparison with layered lithium cobalt oxide (LiCoO_2_, LCO), lithium nickel oxide (LiNiO_2_, LNO), and related systems, the LMO reveals many intrinsic features such as low cost, wide abundance of resources, environmental benignity, high working potential, excellent safety characteristics, and competitive theoretical capacity of approximately 148 mAh·g^−1^ [4,5,6,7]. Nonetheless, the major drawback is that the stoichiometric LiMn_2_O_4_ spinel suffers from severe capacity fading during electrochemical charging/discharging processes [8,9]. This limits its cycle ability along with the rate performance and prevents its broad commercialization. The decrease of capacity is generally attributed to the phase transition occurring in the LMO spinel at room temperature owing to Jahn-Teller distortion of high spin Mn^3+^ ions [10,11,12], and even more importantly, the increased surface reactivity between electrolyte and highly delithiated cathode material, leading to dissolution of manganese in the electrolyte [13,14]. To overcome the above problems, many research efforts have been made so far. One method is to introduce a heterogeneous atom into the LMO structure [15,16,17,18,19,20]. The other way is to modify the surface of the spinel [21,22,23,24,25,26,27]. A coating layer on the spinel particles can reduce the contact area of electrode/electrolyte interface and suppress the dissolution of manganese. Furthermore, it has been reported that nanostructured LMO can present enhanced performance [28,29,30,31].

Although there are a lot of doping modification methods described in the literature, most of them are not compounded (cation or anion only). Accordingly, co-doping still remains worth studying. A very interesting approach, which could improve the structural and chemical properties of LiMn_2_O_4_ spinel material, thereby leading to the promotion of cycling stability, involves the synergetic substitution of nickel and sulfur [32,33]. It is believed that sulfur replacing oxygen in the spinel structure not only reduces the octahedral symmetry, and as a consequence suppresses the phase transition near room temperature, but also increases the capacity of the material as well as improves the coulombic efficiency [20,34]. On the other hand, stabilization of the spinel structure and an increase of its chemical stability can also be performed by partial substitution of the Mn^3+^ ions by other transition metal ions, like Ni [35]. It was reported that a LiMn_2_O_4_ spinel-based electrode, in which a small amount of manganese was substituted by another 3d metal, shows improved cycling performance and can supply a higher energy density than the common lithium manganese spinel by shifting the voltage profile to a higher potentials as new voltage plateaus corresponding to Ni^2+^/Ni^3+^ and Ni^3+^/Ni^4+^ redox couples are introduced [36,37]. In this work we report the synthesis of nanostructured Ni and S doped lithium manganese oxides using a xerogel-type sol-gel method [27,34,38,39]. The aim concerning this study was to find the best compromise among chemical composition and physicochemical properties as well as electrochemical characteristic of the prepared materials. It was discovered that nickel and sulfur substitution can enhance capacity retention and the charge and discharge performance of the LMO cathode under the high current rates.

## 2. Materials and Methods

A xerogel-type sol-gel method was employed to synthesize LiMn_2−*x*_Ni*_x_*O_4–*y*_S*_y_* (LMNOS) spinel materials (0.1 ≤ *x* ≤ 0.5 and *y* = 0.01). In the first step, CH_3_COOLi·2H_2_O, (CH_3_COO)_2_Mn·4H_2_O, and (CH_3_COO)_2_Ni·4H_2_O, in appropriate molar ratio, were dissolved together in distilled water while stirring. Then, (NH_4_)_2_S (20 wt %) and NH_3_·H_2_O (25 wt %) as the alkalizing agent were slowly added into the solution. All the syntheses were conducted under constant flow of argon to prevent uncontrolled oxidation of the Mn^2+^ ions. In the second step, condensation of the formed sols was performed at 90 °C for three to four days under ambient pressure in air. Finally, the obtained xerogels were calcined in a muffle furnace in air at 300 °C for 24 h and afterwards at 650 °C for 6 h. The high-temperature calcination was required to receive improved structural and electrical properties of the spinels and was followed by quenching. The heating rates for the calcinations processes were 1 °C·min^−1^ and 5 °C·min^−1^ respectively.

The X-ray powder diffraction (XRD) was conducted to investigate the crystal structure of the resulting materials using BRUKER D2 PHASER diffractometer (Bruker, Billerica, MA, USA) with Cu Kα radiation (λ = 0.154184 nm) at an operating current of 10 mA and voltage of 30 kV. The diffraction patterns were recorded in the 2θ range of 10° to 80° with a step of 0.02°. To identify the phase composition of the samples, structural data from the International Centre for Diffraction Data (ICDD) was used. The average crystallite size was estimated from the integral width of (111) reflection of the cubic spinel using Scherrer’s equation. The elemental analysis on selected test areas of the synthesized materials was provided by transmission electron microscopy (TEM) using FEI TECNAI TF20 X-TWIN (FEI, Hillsboro, OR, USA) high-resolution microscope operating at 200 kV and equipped with an energy dispersive X-ray (EDX) detector (EDAX, Mahwah, NJ, USA). The textural properties were characterized by N_2_ adsorption-desorption measurements performed at about −196 °C on a Micromeritics 3Flex surface area analyzer (Micromeritics, Norcross, GA, USA) after sample pre-treatment. The specific surface area was calculated according to the Brunauer-Emmett-Teller (BET) method. To determine the pore size distribution and estimate a pore volume and an average pore diameter the Barrett-Joyner-Halenda (BJH) method was applied. To gain knowledge about the phase transition, the differential scanning calorimetry (DSC) experiments were carried out on a Mettler-Toledo 821^e^ instrument equipped with intracooler Haake (Mettler-Toledo, Columbus, OH, USA). Every time, approximately 12 mg of each sample was placed in aluminum crucible and measured in the temperature range of −20 to +50 °C with a heating and cooling rate equal to 10 °C min^−1^ under constant flow of argon (80 mL·min^−1^) (Air Products, Allentown, PA, USA). The electrical conductivity (EC) was studied using the four-probe ac method at 33 Hz within the temperature range of −20 to +40 °C. The powder samples were put between the parallel gold, circular electrodes in a glass tube and pressed by a screw-press until the measured resistance of the sample remains unchanged. The electrical conductivity complies with the Arrhenius law σ = σ_0_∙exp(−*E*_a_/(*k*_B_∙*T*)) where σ_0_ is the pre-exponential factor, *E*_a_ is the activation energy, and *k*_B_, the Boltzmann constant. The slope of the plot in the lnσ *vs.* 1000 T^−1^ coordinates enabled the evaluation of the activation energy.

The electrochemical performance of the synthesized spinels was examined using R2032 coin-type cells. The Li/Li^+^/LMNOS cells were assembled in an argon-filled glove box (MBraun Unilab Plus workstation MBraun, Garching, Germany) with both H_2_O and O_2_ levels less than 0.1 ppm. The cathodes were fabricated by mixing the 80 wt % of active material with 10 wt % of carbon black, used as conductive agent, and 10 wt % of polyvinylidene fluoride (PVDF) binder in *N*-methyl-2-pyrrolidone (NMP) solvent. The prepared slurry was stirred for 24 h and then coated on an aluminum foil to form the working electrodes with 12 mm in diameter. The typical loading of active materials in the assembled cells was around 2.21 mg·cm^−2^. As a negative electrode, a metallic lithium foil was used. Both electrodes were separated by a microporous polypropylene film (Celgard 2325) and a porous glass microfiber filters (Whatman GF/F). The electrolyte was a 1 M solution of lithium hexafluorophosphate (LiPF_6_) in a mixture of ethylene carbonate (EC) and diethyl carbonate (DEC) at a volume ratio of 1:1. The galvanostatic charge and discharge tests (CELL TEST) were run at different *C* rates using ATLAS 0961 MBI multichannel battery tester at room temperature. Cut-off voltages were 4.8 and 3.5 V for the charge and discharge processes, respectively. The electrochemical impedance spectroscopy (EIS), as well as cyclic voltammetry (CV) were conducted on a potentiostat/galvanostat AUTOLAB PGSTAT302N/FRA2 (Metrohm Autolab, Utrecht, The Netherlands). The EIS measurements were made at 3.75 V by applying an alternating current signal of 0.01 V amplitude in the frequency range from 100 kHz to 0.1 Hz. The impedance data was fitted using Nova 1.8 Autolab software based on the Boukamp model. The CV scans were performed at a scan rate of 0.05 mV·s^−1^ in the potential range of 3.5 to 4.8 V, starting from an open circuit voltage (OCV).

## 3. Results and Discussion

**Figure 1:**
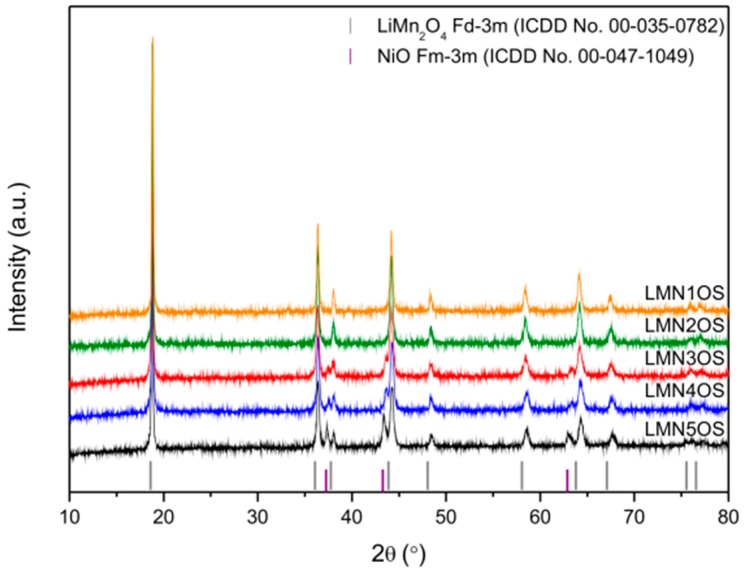
X-ray diffraction patterns of LiMn_2−*x*_Ni*_x_*O_3.99_S_0.01_ spinels calcined at 650 °C.

Figure 1 shows the X-ray diffraction patterns of the prepared LiMn_2−*x*_Ni_*x*_O_4−*y*_S_*y*_ (LMNOS) spinel materials calcined at 650 °C in which 0.1 ≤ *x* ≤ 0.5 and *y* = 0.01. The well-developed, strong, and narrow reflections depict that all products are highly crystallized. For the LiMn_1.5_Ni_0.5_O_3.99_S_0.01_ (LMN5OS), LiMn_1.6_Ni_0.4_O_3.99_S_0.01_ (LMN4OS), and LiMn_1.7_Ni_0.3_O_3.99_S_0.01_ (LMN3OS) samples, the best fit of the XRD patterns was achieved using two phase system: LiMn_2_O_4_ (ICDD No. 00-035-0782) and NiO (ICDD No. 00-047-1049). As for the LiMn_1.8_Ni_0.2_O_3.99_S_0.01_ (LMN2OS) and LiMn_1.9_Ni_0.1_O_3.99_S_0.01_ (LMN1OS) samples, all the diffraction peaks were indexed to the cubic LiMn_2_O_4_ spinel structure (ICDD No. 00-035-0782) with Fd-3m space group, in which lithium ions occupy the 8a sites, manganese ions are located in the 16d sites, and oxygen ions in the 32e sites. No trace of an impurity phase like NiO is observable in these patterns, indicating the formation of single-phase spinel compounds in the 0.1 ≤ *x* ≤ 0.2 Ni substitution range. The lattice parameters of the synthesized materials were calculated from the XRD data (Table 1). The lattice constants for LMNOS samples are slightly lower than for LMO or LMOS1 samples [34] which proves nickel substitution for manganese in the spinel structure and is consistent with the previous studies published by *inter alia* D. H. Park *et al.* [40]. The decrease in the lattice parameter of the modified samples is connected to the increase of average oxidation state of manganese due to Ni substitution and is caused directly by the decline in the amount of Mn^3+^ ions with the higher ionic radii than Mn^4+^ ions. The average crystallite size of the LMNOS powders was estimated using Scherrer’s equation and summarized in Table 1.

**Figure 2:**
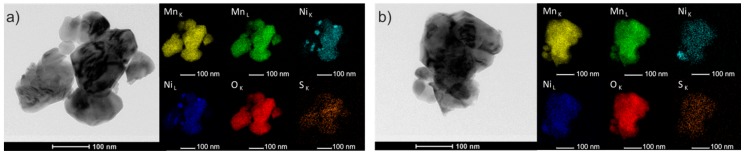
Transmission electron microscopy images and elemental mapping of (**a**) LMN5OS sample; and (**b**) LMN1OS.

To confirm the presence of nickel and sulfur in the obtained spinel materials (LMN5OS and LMN1OS) we present the TEM images of selected test area and the energy dispersive X-ray spectroscopy (EDS) mapping of individual elements analysis (Figure 2). As shown, all observed elements (for both samples) have homogeneous distribution and there is no significant agglomeration of particular components. These results prove that Ni and S atoms were doped uniformly into the LMO spinel via the sol-gel process. Additionally, the EDS maps of Ni for LMN5OS material display some grains of NiO, which is compatible with the formation of single-phase spinel compounds only in the 0.1 ≤ *x* ≤ 0.2 Ni substitution range by the sol-gel method. Obviously, lithium was not revealed in this study as it is beyond the EDS detection range.

**Figure 3:**
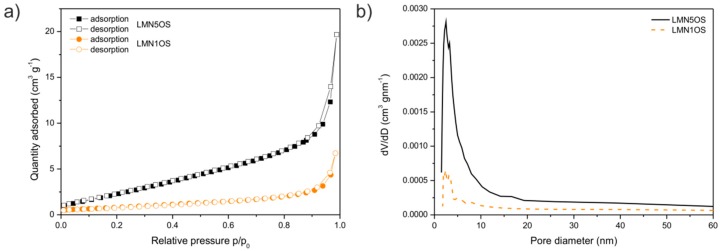
(**a**) N_2_ adsorption-desorption isotherms; and (**b**) the BJH (Barrett-Joyner-Halenda) pore size distributions of LMN5OS and LMN1OS systems.

The nitrogen adsorption-desorption isotherms of LMN5OS and LMN1OS samples are illustrated in Figure 3a. The LMN1OS spinel in comparison with LMN5OS material demonstrates a decreased amount of nitrogen adsorption and desorption. In spite of the fact that the isotherms’ shape may imply that the powders are mostly macroporous (the hysteresis loops are extremely narrow), the BJH pore size distribution analysis (Figure 3b), based on the adsorption branch data, exhibited significant volumes of mesopores in the diameter range of 1.5 to 20 nm with the peak pore size centered at around 2.5 nm for both LMNOS systems. In accordance with the isotherms, the textural properties such as surface area (*S*_BET_), pore volume (*V*_p_), and average pore diameter (*D*_p_) of all obtained materials were calculated and collected in Table 1. It is noticeable that the *S*_BET_ value for LMNOS spinels diminishes with the smaller nickel content. Generally speaking, the raise in the BET surface area will extend the contact region between the electrode and liquid electrolyte. Hence, the lowest specific area for the prepared LMN1OS sample greatly contributes to the observed enhanced cycling stability of the electrode (Figure 6d).

**Figure 4:**
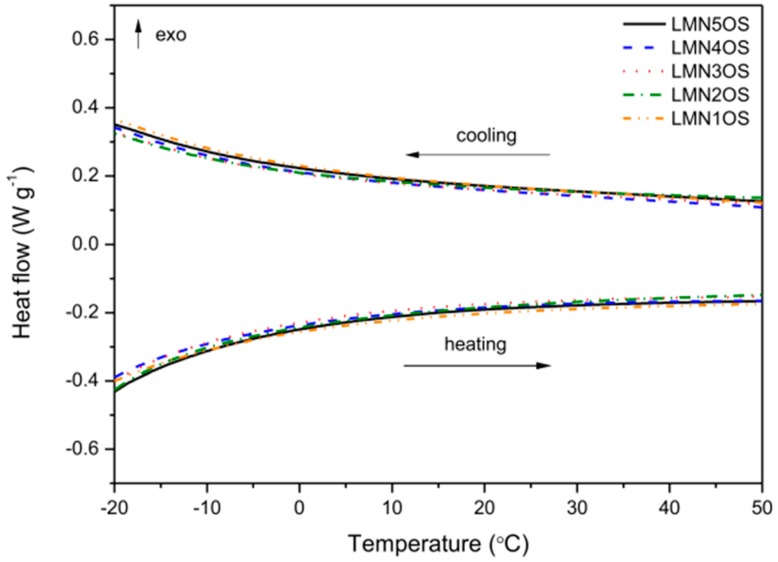
Differential scanning calorimetry results of Ni and S doped LiMn_2_O_4_ materials calcined at 650 °C.

Figure 4 displays the results of the DSC experiments of the nickel and sulfur doped LiMn_2_O_4_ materials. The differential scanning calorimetry curves recorded for all synthesized products present a similar behavior. In fact, no differences in the results of DSC analyses with the change of nickel content in the LiMn_2−*x*_Ni*_x_*O_3.99_S_0.01_ spinel structure were recognized. Furthermore, no heat effects were noticed during heating and cooling for all spinels, thereby indicating that they do not undergo a reversible cubic-orthorhombic phase transition, characteristic for LMO material [41]. On the whole, the subtle deviation of the LiMn_2_O_4_ stoichiometry, resulting in this case from the substitution of manganese and oxygen with nickel and sulfur respectively, stabilizes the spinel structure and suppresses the phase transition which is reflected in the thermal behavior of the compounds.

**Figure 5:**
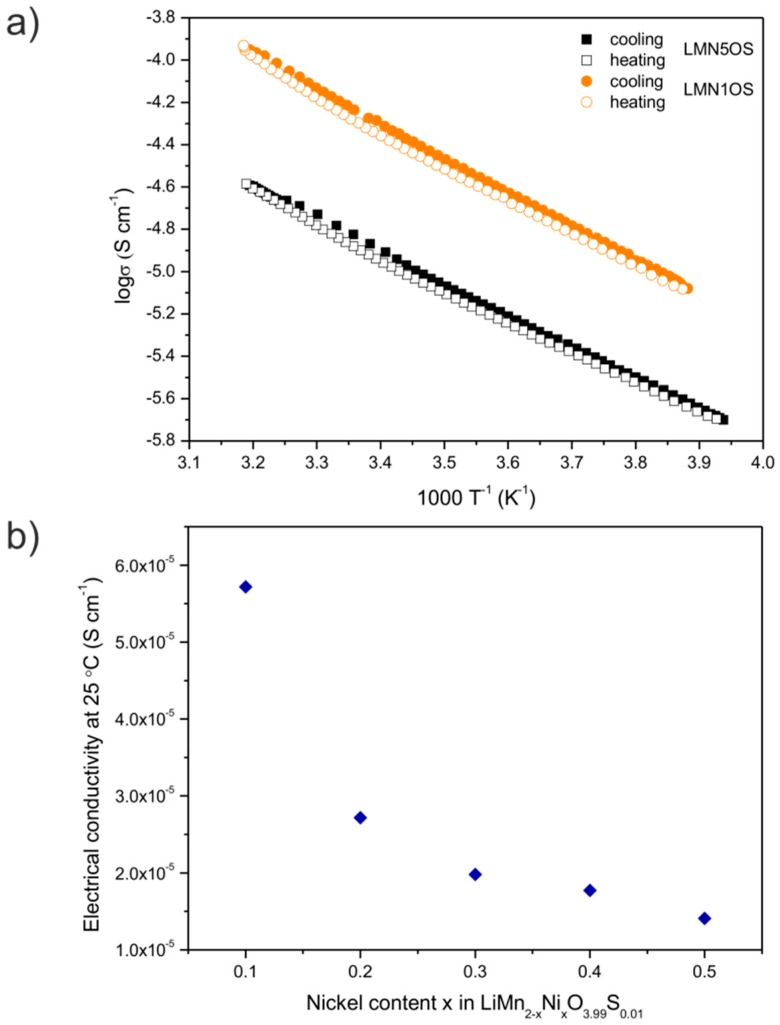
(**a**) The Arrhenius plots of logσ *vs.* 1000 T^−1^ of LiMn_1.5_Ni_0.5_O_3.99_S_0.01_ and LiMn_1.9_Ni_0.1_O_3.99_S_0.01_ spinel materials; and (**b**) the electrical conductivity estimated at room temperature as a function of nickel content *x* in LiMn_2−*x*_Ni*_x_*O_3.99_S_0.01_.

The dependence of electrical conductivity of the synthesized LiMn_2−*x*_Ni*_x_*O_3.99_S_0.01_ (*x* = 0.5 and 0.1) spinels *vs.* reciprocal temperature (1000 T^−1^) is presented in Figure 5a. The Arrhenius relations of the electrical conductivity show that the conduction process is thermally activated due to the semiconducting nature of these materials over the studied temperature range. The obtained results of electrical conductivity for LMNOS samples do not depict an anomalous behavior near room temperature as it was reported for the stoichiometric LiMn_2_O_4_ spinel [34]. The linear dependencies represented in the plots assure that no structural changes occur in the temperature range of −20 to +40 °C which is in good accordance with the DSC results (Figure 4). Thus, we can maintain that the introduction of nickel and sulfur in the LiMn_2_O_4_ spinel structure led to the suppression of the unfavorable phase transition. The estimated values of electrical conductivity at around 25 °C and the activation energy in the −20 to +40 °C temperature range are gathered in Table 2. These values are typical for small-polaron conduction mechanism in a mixed-valent system [42,43]. The σ measured at room temperature is affected by nickel substitution, as illustrated in Figure 5b. It was indicated that the decrease of Ni content in the spinel structure gives rise to electrical conductivity. What is more, the electrical conductivity for the LMN1OS sample is higher than for LMO and LMOS1 materials. We can also remark that the activation energy remains almost constant (around 0.31 eV), which is nevertheless lower than for LMO and LMOS1 materials [34].

**Figure 6:**
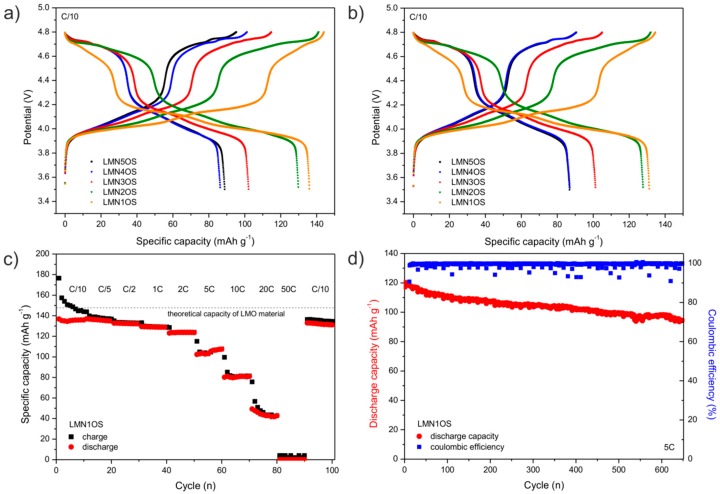
Galvanostatic charge-discharge voltage profiles for (**a**) the tenth; and (**b**) hundredth cycle of the LMNOS cathode materials at *C*/10 current rate; and (**c**) change in specific charge-discharge capacity as a function of cycle at various *C* rates; and (**d**) long cycling performance at 5*C* of LMN1OS electrode.

Figure 6a,b compare charge-discharge voltage profiles for the tenth and hundredth cycle of the Li/Li^+^/LMNOS cells at *C*/10 rate at room temperature. It was found that all charge-discharge curves can be divided into two regions at around 4.1 V (major region) and 4.7 V (minor region) which reflect the electrochemical behavior of LiMn_2_O_4_ as well as LiMn_1.5_Ni_0.5_O_4,_ and correspond to lithium ions extraction/insertion into the cubic spinel structure. Each region exhibits two distinctive plateaus. The two plateaus at ~4.1 V are attributed to the oxidation/reduction of manganese (Mn^3+^/Mn^4+^ redox couple), while the two plateaus at ~4.7 V originate from oxidation/reduction of nickel (Ni^2+^/Ni^3+^ and Ni^3+^/Ni^4+^ redox couples). The rate performance of LMNOS powders was also investigated. The fabricated cathode materials were subjected to 10 sets of 10 cycles at the different *C* rates ranging from *C*/10 to 50*C* at room temperature. As expected, the charge and discharge capacity decreased with the increase of current rate for all lithium cells with nickel and sulfur doped spinels as cathodes. Nevertheless, it is worth noting that very small declines in the capacity are observed during cycling within a single set. The specific charge-discharge capacity of the assembled Li-ion cell with LMN1OS material *vs.* cycle number is displayed in Figure 6c. The first discharge capacity of the LiMn_1.9_Ni_0.1_O_3.99_S_0.01_ cathode material is 136.8 mAh·g^−1^ (*C*/10), which constitutes about 92% of the theoretical capacity of the undoped LMO, and it decreases as the current rate increases to 134.9 (*C*/5), 132.1 (*C*/2), 128.7 (1*C*), 123.6 (2*C*), 107.5 (5*C*), 81.5 (10*C*), and 42.8 mAh·g^−1^ (20*C*), respectively. Even with the further increase of charge-discharge rate to 50*C*, the capacity can be approximately recovered when the current density is returned to *C*/10. The reversible capacity of 131.2 mAh·g^−1^ was then retained. In addition, the cycle performance of LMN1OS spinel was evaluated with long galvanostatic cycling tests at the current density of 5*C* (740 mA·g^−1^) at room temperature. Figure 6d presents the dependence of the discharge capacity on cycle number. The initial discharge capacity of the sample is 117.3 mAh·g^−1^ which declines to 94.5 mAh·g^−1^ after 650 cycles. Thus, the total capacity retention for the LMN1OS electrode is 80.6%. Apart from the cycling stability, Figure 6d indicates coulombic efficiencies that range from 91% to 100%. In short, the presented results demonstrate excellent electrochemical performance of the LiMn_1.9_Ni_0.1_O_3.99_S_0.01_ cathode material, including high rate capability, and outstanding capacity retention. This characteristic of nickel and sulfur spinel synthesized in the following study may be attributed to the highly crystalline, nanostructured, and, most importantly, structurally stable nature of the material with improved electrical properties.

**Figure 7:**
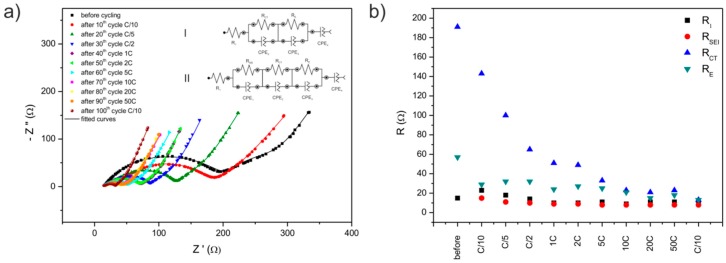
Nyquist plots for Li/Li^+^/LiMn_1.9_Ni_0.1_O_3.99_S_0.01_ cell with the equivalent circuits used to model (**a**) the EIS (electrochemical impedance spectroscopy) spectra; and (**b**) the changes of EIS parameters.

The electrochemical impedance spectra of the Li/Li^+^/LMN1OS cell (Figure 7a) were obtained at 3.75 V before cycling and after each set of 10 cycles (Figure 6c) at room temperature. The resulting Nyquist plots show an unusual trend for compounds of the spinel group. They are composed of the three depressed semicircles in the high-to-low frequencies and a straight line in the low frequency range. This phenomenon, however, has already been reported in the literature [44]. The impedance spectra can be interpreted on the basis of the proposed equivalent circuit (inset II of Figure 7a), the same for all curves, except the first one recorded before cycling (inset I of Figure 7a). In these circuits, R_1_ refers to the uncompensated resistance of liquid electrolyte and the resistance between the electrode and the current collector. The *R*_1_ corresponds to the high frequency intercept at the real axis. *R*_SEI_ and *R*_CT_ are the resistances which are used to model two depressed semicircles. The first depressed semicircle (at the high frequency region) is ascribed to lithium ion diffusion through the passivation layer (SEI), and the second depressed semicircle (at the high-to-medium frequency region) is assigned to the charge transfer reaction of electrode material. The capacitance of the SEI film and the capacitance of the double layer are represented by the constant phase elements (CPE), CPE_1_ and CPE_2_, respectively. *R*_E_ and CPE_3_ stand for the electronic resistance of the material and the associated capacitance used to characterize the electronic properties of the material and model the third depressed semicircle (at the medium-to-low frequency region). Another CPE element (CPE_4_) is responsible in the proposed circuit for modelling the line at the low frequencies. As a matter of fact, this is a Warburg-type element (0.5 < *N* < 1) which is attributed to the lithium ion diffusion. The values of each resistor from the fitted circuits are given in Table 3. A significant decrease of *R*_CT_ resistance during cell cycling (Figure 7b) is a direct indication of an increase of electrical transfer in the material resulting in a decrease of the cell polarization, thereby allowing a partial compensation of capacity fading; therefore, the LMN1OS electrode reveals improved overall electrochemical performance. Furthermore, it was shown that the increase of charge-discharge rate to 50*C* does not cause the SEI destruction, as the *R*_SEI_ remains constant. Some changes, however, are induced in the *R*_CT_ and *R*_E_ values, after applying 50*C* rate, as presented in Figure 7b.

**Figure 8:**
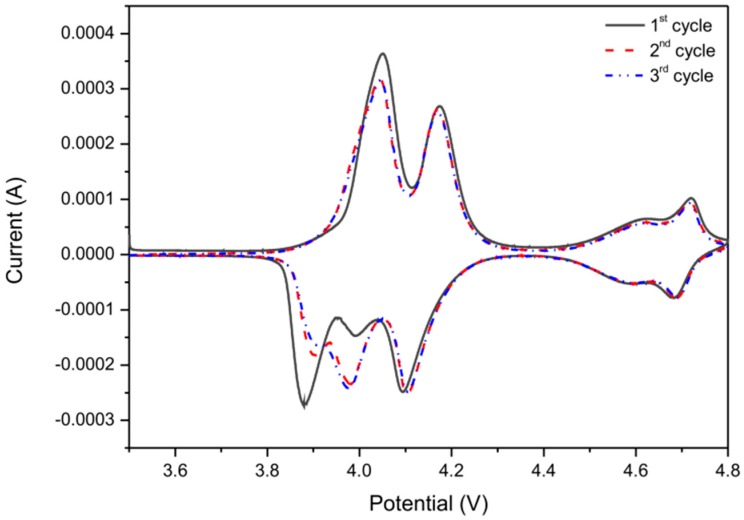
The cyclic voltammetry curves for a LMN1OS electrode with a scan rate of 0.05 mV·s^−1^.

Three subsequent cyclic voltammetry (CV) curves of LMN1OS electrode, measured at room temperature in the range from 3.5 to 4.8 V, are depicted in Figure 8. These voltammograms demonstrate two main regions of electrochemical activity of the sample which are related to the four pairs of reversible oxidation and reduction current peaks at 4.05, 4.17, 4.62, 4.72 V and 3.99, 4.09, 4.59, 4.69 V respectively. The major doublet redox peaks at around 4.1 V originate from the Mn^3+^/Mn^4+^ redox couple, whereas the smaller redox peaks at around 4.7 V are ascribed to the Ni^2+^/Ni^3+^ and Ni^3+^/Ni^4+^ redox couples. As presented in the literature, for ordered LiMn_1.5_Ni_0.5_O_4_ spinel the ~4.1 V peaks are not observed from the CV because oxidation states of Ni and Mn are +2 and +4 respectively [45]. In this case, the appearance of ~4.1 V peaks signal the existence of Mn^3+^ ions which are mainly responsible for the capacity of the LMN1OS material. The results of the CV are in good agreement with those preceding galvanostatic charge-discharge experiments (associated plateaus in the charge/discharge profiles in Figure 6a,b). Besides the well-defined and well-known redox peaks, a peak at around 3.88 V is observed. It may be related to the structure ordering of nickel and sulfur doped LMO spinel, as the peak is gradually converting during cycling. This behavior of LMN1OS spinel can be also concluded from Figure 6c (the first ten cycles).

## 4. Conclusions

Nickel and sulfur co-doped LiMn_2_O_4_ cathode materials were successfully synthesized by the xerogel-type sol-gel process. Based on this method, nanosized materials with spinel structure, homogeneous distributions of elements, and various nickel content were obtained. However, the formation of single-phase spinel compounds is possible in the 0.1 ≤ *x* ≤ 0.2 Ni substitution range. Due to the introduction of Ni and S in the LMO spinel structure, the unfavorable phase transition around room temperature was diminished. What is more, the additive of nickel in the LiMn_2_O_4_ affected the electrical properties of the spinel. It was indicated that the decrease of nickel content in the spinel structure gives rise to electrical conductivity at room temperature, while the activation energies of conduction remained constant but still lower than for the LMO material. The electrochemical studies performed with the synthesized materials delivered high capacity and excellent cycling behavior of the LiMn_1.9_Ni_0.1_O_3.99_S_0.01_ cathode material (the first discharge capacity of the LMN1OS cathode is about 10 mAh·g^−1^ higher than for the LMO sample obtained by the same sol-gel procedure [34]). Additionally, it was demonstrated that the nickel and sulfur doped spinel show outstanding rate capability, very good capacity retention, and reversibility in comparison to the stoichiometric spinel. It was found that the loss of initial capacity for the LMN1OS sample was only around 19% after 650 cycles at 5*C* current density. On the basis of our results it can be pointed out that the effect of synergetic substitution of lithium manganese oxide spinel with Ni and S is an efficient way to promote its structural stability and electrochemical performance in Li-ion cells.

## Figures and Tables

**Table 1:** Chemical composition, lattice constant, average crystallites size, and textural properties of nanostructured LiMn_2−*x*_Ni*_x_*O_4−*y*_S*_y_* spinels in which 0.1 ≤ *x* ≤ 0.5 and *y* = 0.01.

Sample	Nominal Composition	Lattice Constant (nm)	Average Crystallites Size (nm)	Surface BET Area (m^2^·g^−1^)	Pore Volume (cm^3^·g^−1^)	Average Pore Diameter (nm)
LMN5OS	LiMn_1.5_Ni_0.5_O_3.99_S_0.01_	0.8181	42	10.9	0.030	11
LMN4OS	LiMn_1.6_Ni_0.4_O_3.99_S_0.01_	0.8169	40	8.7	0.041	19
LMN3OS	LiMn_1.7_Ni_0.3_O_3.99_S_0.01_	0.8183	36	7.9	0.030	19
LMN2OS	LiMn_1.8_Ni_0.2_O_3.99_S_0.01_	0.8172	47	3.5	0.012	13
LMN1OS	LiMn_1.9_Ni_0.1_O_3.99_S_0.01_	0.8149	48	3.1	0.010	13

**Table 2:** Electrical properties of the synthesized spinels.

Sample	Activation Energy (Cooling) (eV)	Activation Energy (Heating) (eV)	Electrical Conductivity at Around 25 °C (Cooling) (10^−5^·S·cm^−1^)	Electrical Conductivity at Around 25 °C (Heating) (10^−5^·S·cm^−1^)
LMN5OS	0.30	0.30	1.46	1.35
LMN4OS	0.30	0.30	1.84	1.71
LMN3OS	0.30	0.30	2.12	1.84
LMN2OS	0.32	0.32	2.84	2.59
LMN1OS	0.32	0.32	5.97	5.46

**Table 3:** Parameters of EIS measurements (calculated values of resistors in proposed equivalent circuits) for LMN1OS electrode.

	*R*_1_ (Ω)	*R*_SEI_ (Ω)	*R*_CT_ (Ω)	*R*_E_ (Ω)
before cycling	15	-	191	57
after 10th cycle *C*/10	23	15	143	29
after 20th cycle *C*/5	18	11	100	32
after 30th cycle *C*/2	14	10	65	32
after 40th cycle 1*C*	10	9	51	24
after 50th cycle 2*C*	10	9	49	27
after 60th cycle 5*C*	11	8	33	25
after 70th cycle 10*C*	9	8	23	21
after 80th cycle 20*C*	11	8	21	15
after 90th cycle 50*C*	11	8	23	18
after 100th cycle *C*/10	11	8	13	13
